# A Hyper-Viscoelastic Continuum-Level Finite Element Model of the Spinal Cord Assessed for Transverse Indentation and Impact Loading

**DOI:** 10.3389/fbioe.2021.693120

**Published:** 2021-08-12

**Authors:** Aleksander Rycman, Stewart McLachlin, Duane S. Cronin

**Affiliations:** Department of Mechanical and Mechatronics Engineering, University of Waterloo, Waterloo, ON, Canada

**Keywords:** spinal cord, hyper-viscoelastic material model, finite element method, indentation, impact loading

## Abstract

Finite Element (FE) modelling of spinal cord response to impact can provide unique insights into the neural tissue response and injury risk potential. Yet, contemporary human body models (HBMs) used to examine injury risk and prevention across a wide range of impact scenarios often lack detailed integration of the spinal cord and surrounding tissues. The integration of a spinal cord in contemporary HBMs has been limited by the need for a continuum-level model owing to the relatively large element size required to be compatible with HBM, and the requirement for model development based on published material properties and validation using relevant non-linear material data. The goals of this study were to develop and assess non-linear material model parameters for the spinal cord parenchyma and pia mater, and incorporate these models into a continuum-level model of the spinal cord with a mesh size conducive to integration in HBM. First, hyper-viscoelastic material properties based on tissue-level mechanical test data for the spinal cord and hyperelastic material properties for the pia mater were determined. Secondly, the constitutive models were integrated in a spinal cord segment FE model validated against independent experimental data representing transverse compression of the spinal cord-pia mater complex (SCP) under quasi-static indentation and dynamic impact loading. The constitutive model parameters were fit to a quasi-linear viscoelastic model with an Ogden hyperelastic function, and then verified using single element test cases corresponding to the experimental strain rates for the spinal cord (0.32–77.22 s^−1^) and pia mater (0.05 s^−1^). Validation of the spinal cord model was then performed by re-creating, in an explicit FE code, two independent *ex-vivo* experimental setups: 1) transverse indentation of a porcine spinal cord-pia mater complex and 2) dynamic transverse impact of a bovine SCP. The indentation model accurately matched the experimental results up to 60% compression of the SCP, while the impact model predicted the loading phase and the maximum deformation (within 7%) of the SCP experimental data. This study quantified the important biomechanical contribution of the pia mater tissue during spinal cord deformation. The validated material models established in this study can be implemented in computational HBM.

## Introduction

Computational finite element (FE) models can help to identify the salient mechanical properties that drive soft tissue behaviour in response to loading, to examine potentially injurious loading scenarios (Schmitt et al., 2019). In the context of spinal cord injury (SCI), the primary challenge with FE modelling lies in biofidelity, the ability of the model to accurately reproduce the behaviour of the simulated tissues (Cronin, 2011; Yang, 2017). Previous research has established that material properties and model validation are most important to the biofidelity of a FE model to predict SCI (Jones and Clarke, 2018). This is challenging due to the complexity of the spinal cord, consisting of multiple neural tissues, including the spinal cord parenchyma (white and gray matter), pia-arachnoid mater complex, and other connective tissues (Standring et al., 2009). Further, modeling spinal cord tissues in FE models is difficult due to the non-linear behaviour of the connected tissues, which includes hyperelastic and viscoelastic tissue response under deformation (Bilston, 2011).

There are several studies that have investigated spinal cord FE models with varying degrees of complexity in terms of the included tissues and material models used to represent the tissues (Ichihara et al., 2001; Wilcox et al., 2003; Wilcox et al., 2004; Czyz et al., 2008; Li and Dai, 2009; Czyz et al., 2011; Persson et al., 2011; Jannesar et al., 2016; Bailly et al., 2020). Although some studies have proposed non-linear material models (Jannesar et al., 2020), the associated models typically involve very small mesh sizes (e.g., <0.5 mm) that are prohibitive for current state-of-the-art HBM (Schwartz et al., 2015; Kimpara et al., 2016; Östh et al., 2017). In contrast, models utilizing coarse meshes often incorporate simplified elastic material properties (Greaves et al., 2008) and have not been evaluated in terms of model biofidelity for impact scenarios (Kimpara et al., 2006; Henao et al., 2016, Henao et al., 2018; Diotalevi et al., 2020). Importantly, the existing models also lack hierarchical assessment and robust validation that are needed prior to integration in HBM.

The nature of SCI is mostly dynamic; rapid deformation of the spinal cord tissues is caused either by hyper-rotation of the spinal column, or by impact from a bony fragment resulting from vertebral fracture (Mattucci et al., 2019). In this case, to validate dynamic deformation of any spinal cord FE model requires dynamic experimental data; however, this type of tissue-level validation data is limited. Fradet et al. (2016) investigated response of the porcine spinal cord-pia mater complex (SCP) complex to transverse indentation by a small cylinder (indenter) at a quasi-static and high rate. Another *ex-vivo* test included a series of impacts on the bovine SCP complex by small impactors (Persson et al., 2009). These tests were performed at impact speeds associated with burst fracture of the vertebral body.

The overarching objective of the current study was to develop non-linear material models applicable to a continuum-level spinal cord model that could be integrated into a contemporary HBM. As a first step, tissue-level experimental data (Jin et al., 2006; Jannesar et al., 2018) was utilized to fit parameters of the non-linear constitutive models. Validation of fitted material models was performed by recreating *ex-vivo* transverse indentation of the porcine SCP complex (Fradet et al., 2016) and *ex-vivo* transverse impact test on the bovine SCP complex (Persson, 2009; Persson et al., 2009). The validation process was independent of the fitting process; the values of the material parameters obtained in the fitting process were not altered in the indentation or impact model. Lastly, the role of the pia mater and its thickness during the dynamic impact of the SCP complex was examined.

## Materials and Methods

### Material Models for the Spinal Cord—Pia Complex

The spinal cord material response was determined from experimental data of unconfined compression of non-human primate spinal white mater (Jannesar et al., 2018) and used to fit isotropic hyperelastic material coefficients using an Ogden constitutive model (Ogden, 1972). Viscoelastic effects were incorporated using a quasi-linear viscoelastic formulation (Fung, 1981; Xu et al., 2008) with normalized Prony series shear moduli (G_i_) and relaxation times (*ß*_i_). The explicit FE software utilized a modified strain energy density Ogden function (Hallquist, 2006) for the deviatoric and hydrostatic components of deformation (Eq. 1). The coefficients *µ*_i_ and *α*_i_ are *i*th Ogden model material constants, λ* are the deviatoric principal stretches, K is the bulk modulus and J is the relative volume.

$$W\left(\lambda_{1},\lambda_{2},\lambda_{3}\right)=\sum_{i=1}^{N}\frac{\mu_{i}}{\alpha_{i}}\left(\lambda_{1}^{*\alpha_{i}}+\lambda_{2}^{*\alpha_{i}}+\lambda_{3}^{*\alpha_{i}}-3\right)+\frac{1}{2}K{\left(J-1\right)}^{2}\;$$ (1)

Equation 1: Modified Ogden strain energy density function including the hydrostatic component.

Stress in the quasi-linear viscoelastic formulation was represented in the form of the Boltzmann hereditary integral including the relaxation modulus (G) and strain rate (Eq. 2).

$$\sigma \left(t\right)=\int_{0}^{t}G\left(t-\tau ,\varepsilon \right)\dot{\varepsilon }\left(t\right)\text{d}\tau \;$$ (2)

Equation 2: Quasi-linear viscoelastic constitutive model.

In quasi-linear viscoelasticity, the relaxation function can be separated into strain-dependant and time-depended parts (Xu et al., 2008) (Eq. 3). The strain-dependant part $\left({\text{σ}}^{\text{E}}\left(\text{ε}\right)\right)$ is derived from the Ogden strain energy density function (Eq. 1); whereas the time-dependant part is formulated as a sum of the Prony series exponential relaxation functions (Eq. 4).

$$G\left(t,\varepsilon \right)=g\left(t\right)\sigma^{E}\left(\varepsilon \right)$$ (3)

Equation 3: Separation of the relaxation function to time-dependent and stress-dependent components.

$$g\left(t\right)=\sum_{i=1\;}^{n}G_{i}e^{-\beta_{i}t}\;$$ (4)

Equation 4: The time-dependent part of the viscoelastic relaxation function formulated with the Prony series.

Finally, substituting Eqs. 3, 4 to Eq. 2, the stress in the quasi-linear viscoelastic material can be expressed in the form:

$$\sigma \left(t\right)=\int_{0}^{t}\sum_{i=1\;}^{n}G_{i}e^{-\beta_{i}\left(t-\tau \right)}\left[\frac{\partial \sigma^{E}}{\partial \varepsilon }\frac{\partial \varepsilon }{\partial \tau }\right]d\tau$$ (5)

Equation 5: Quasi-linear viscoelastic model total stress as a function of time and deformation.

The hyperelastic and viscoelastic coefficients were determined using commercial optimization software (LS-OPT v6.0.0, LST, Livermore, CA). Single element test cases were simulated at each experimental strain rate (i.e., 0.32, 2.83, 25.44, and 77.22 s^−1^) and the material parameters were determined using the curve mapping optimization method (LS-OPT Manual, LST, Livermore, CA). The lowest strain rate data (0.32 s^−1^) was treated as quasi-static and used to fit the hyperelastic function. Three sets of viscoelastic constants (G_i_ and *ß*_i_), with *ß*_i_ values corresponding to the strain rates were used as initial guesses for the fitting algorithm. The normalized shear coefficient (G_i_) values were constrained such that their sum was less than unity, and initial guesses were set to 0.25 with a maximum value of 1.0. The optimization algorithm compared the uniaxial compression response of the single element for each strain rate and the range of strains reported in the experiments (Jannesar et al., 2018). The results of each model were combined using a multi-objective function that gave the results from each strain rate an equal weighting and sought to minimize the difference between the areas under experimental stress-strain curves with the calculated single element response. Specifically, a robust curve mapping algorithm (LS-OPT Manual, LST, Livermore, CA) was used to compare the predicted and experimental responses. Convergence was achieved when the difference between the curves was less than 0.5%. The pia mater mechanical response under deformation exhibits a typical response for collagen-rich tissue with distinct toe, linear, traumatic, and post-failure regions. Tissue-level tensile experimental test data (Jin et al., 2006) was utilized to fit the hyperelastic coefficients of the Ogden model for the pia mater. Quasi-static (0.05 s^−1^) experimental stress-strain curves were used to fit a single term Ogden model. Model coefficients were found using GNU Microsoft Excel solver (Microsoft, Redmond, WA) with an optimization target to maximize the coefficient of determination. The fit was confirmed by comparing a single element verification model to the experimental data.

### Transverse Indentation of the Porcine Spinal Cord-Pia Mater Complex

**FIGURE 1:**
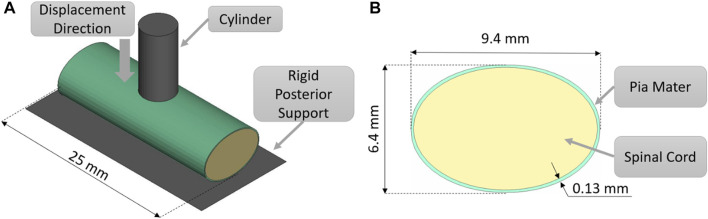
**(A)** Indentation test simulation setup, isometric view. **(B)** Transverse cross-sectional view of the spinal cord-pia mater complex with the dimensions used in the indentation simulations.

The indentation validation case was simulated (Figure 1) by recreating *ex-vivo* experimental setup where porcine cervical SCP complex specimen was indented by a small cylinder (Fradet et al., 2016). A computational FE model was established to recreate the experimental setup, consisting of the porcine tissue specimen, 25 mm in length, a rigid posterior support, and a cylindrical indenter with a diameter of 5 mm (cross-sectional area of 19.6 mm^2^). Anterior-posterior and lateral dimensions of the porcine spinal cord were adopted from experimental measurements of the cord cross-section (Fradet et al., 2016). Since the impactor was relatively small compared to the specimen dimensions, a smaller mesh was required for the spinal cord to accurately predict loading at the boundary between spinal cord and indenter. The indentation model was simulated using three mesh sizes: 0.8, 0.6, and 0.45 mm to establish convergence. The mesh sizes of 0.6 and 0.45 mm converged to a similar force value at 10, 20, 30 40, and 50%, transverse compression. However, some contact instabilities, attributed to mesh sliding on the indenter, were observed while using a mesh size of 0.8 and 0.6 mm; therefore, a mesh size of 0.45 mm was used for the simulations in this study. The static and dynamic friction coefficients between the pia mater and the metal impactor were assumed to be 0.1, following a value proposed for brain tissue and metal (Rashid et al., 2012). To achieve stable contact between tissue and cylinder, a pre-load of 0.2 N was applied as in the experiment. Indentation simulations were performed for three strain rates: 0.5, 5, and 50 s^−1^. The resulting force acting on the cylinder from deformation of the SCP complex was compared to experimental data (Fradet et al., 2016). The thickness of the pia mater was 0.13 mm, as previously reported for porcine specimens (Kimpara et al., 2006).

### Transverse Impact on the Spinal Cord-Pia Mater Complex

**FIGURE 2:**
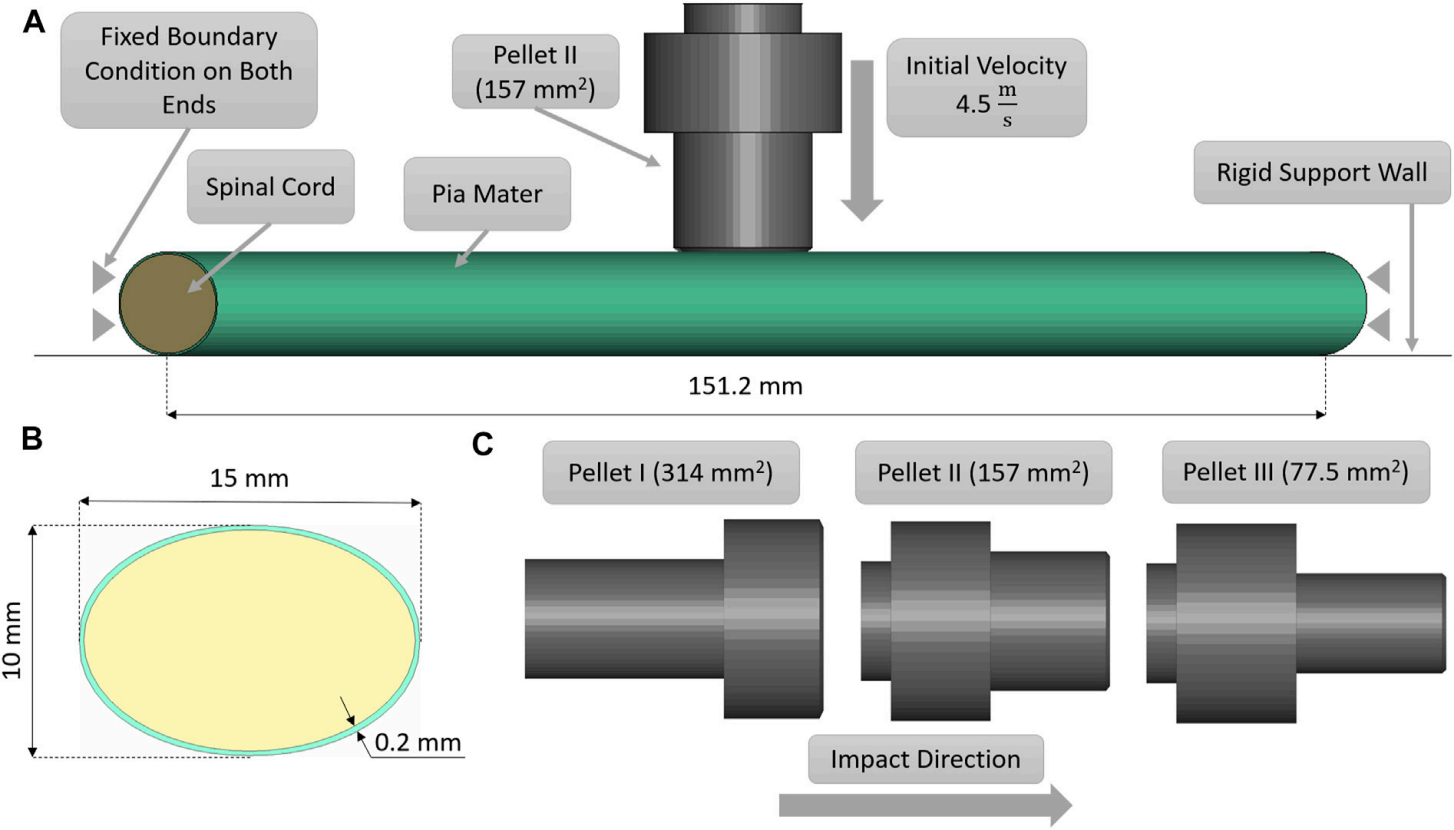
**(A)** Impact test simulation setup with the Pellet II case. **(B)** Sagittal view of the spinal cord-pia mater complex dimensions used in the impact simulations. **(C)** Numerical representations of the three pellet geometries.

Transverse impact on the SCP complex was simulated by replicating *ex-vivo* experimental tests on bovine spinal cord-pia mater specimens. The test specimens were 140 mm long and were subjected to 8% engineering strain preload along the length, followed by a dynamic relaxation period of 60 s. Next, a pellet was accelerated with a pneumatic actuator and impacted the mid-span of the bovine specimen that was resting on a supporting surface. In total, three pellet impacts were conducted at the reported velocity of 4.5 m/s. The trajectories of the pellets (displacement versus time) were reported at 4,500 Hz (Persson, 2009; Persson et al., 2009). A three-dimensional FE model (Figure 2A), which included the cervical nervous tissues (the spinal cord and pia mater), the rigid parts of the support wall, and the three impactors (pellets), was designed based on previously published experimental and computational literature data (Persson et al., 2011). The model did not include spinal nerves as they were trimmed in the experiment. A commercial meshing software (HyperMesh, Altair Engineering, Troy, MI) was used to generate the FE mesh. The spinal cord and pia mater were represented by 37,100 and 9,100 fully integrated hexahedral elements, respectively, with an average element size of 0.8 mm. Mesh convergence was conducted by a simulating model with three average mesh sizes 0.8, 0.4, and 0.2 mm. The maximum deformations of the SCP complex were used with Richardson extrapolation (Roache, 1998) to estimate the 0 mm element solution. It was found that all three mesh sizes were located in the asymptotic convergence region and the percent difference between meshes was less than 1.5%; therefore, an average element size of 0.8 mm was identified as sufficient to model the dynamic behaviour of the SCP complex. In the experiments, the three pellets had the same mass (7 g) but different impact areas: 314 mm^2^ (Pellet I), 157 mm^2^ (Pellet II), and 78,5 mm^2^ (Pellet III) (Figure 2B). For the same impact velocity, decreasing the impact area of the pellets creates a more aggressive insult to the SCP complex and provides a range of validation data that achieve varying levels of strain and strain rate within the tissues.

The static and dynamic friction coefficient between the pia mater and the impactor was 0.1 (Rashid et al., 2012). A velocity sensitivity study was conducted using the maximum, minimum, and average velocities for each pellet. The pellet velocities were calculated based on the initial slope of the experimental pellet trajectory curves to quantify the experimental variability in the context of the reported typical 4.5 m/s pellet velocity. Finally, a thickness sensitivity study was conducted for the pia mater to evaluate the overall effect of the pia mater thickness (0, 0.13, 0.20, and 0.27 mm) on the spinal cord complex compression during impact (Kimpara et al., 2006; Ramo et al., 2018). The proposed pia mater constitutive model for the current study was compared to a widely used linear elastic model (E = 39.3 MPa) (Kimpara et al., 2006).The pia mater thickness and material variations were assessed using the maximum principal strain induced in a volume of the pia mater in the impact zone. The volume considered comprised the diameter of the pellet plus one diameter of the spinal cord on either side of the pellet, which was the highly deformed length of the spinal cord.

### Software and Data Analysis

A commercial meshing software (HyperMesh, Altair Engineering, Troy, MI) was used to generate the geometry and FE mesh for the spinal cord parenchyma and pia mater for both models. Material coefficients for the hyper-viscoelastic model for the spinal cord parenchyma were obtained using commercial optimization software (LS-OPT v6.0.0, LST, Livermore, CA) with an embedded version of a commercial explicit FE solver (LS-DYNA R9.3.1 double-precision). Hyperelastic parameters for the pia mater material model were fitted using GNU Microsoft Excel solver (Microsoft, Redmond, WA). Transverse indentation and impact models were solved using with a commercial explicit FE software (LS-DYNA version R9.3.1 MPP, double-precision, LST, Livermore, CA, on Intel Xeon E5-2,683 2.1 GHz processors). The simulation results that included the displacement of the pellet, the internal energy of the individual tissues, the strain rate history of the spinal cord elements, and contact force between the cylinder and tissue were analyzed using a commercial post-processing software (LS-PrePost version 4.7.9). Fit of the material models were quantified using the coefficient of determination (R^2^), defined as the proportion of variance of the model to the experimental data (Eq. 6).

$$R^{2}=1-\frac{RSS}{TSS}\;\;where:\;RSS=\sum_{i=1}^{N}{\left(y_{\exp,i}-y_{\mathrm{mod}\mathrm{el},i}\right)}^{2};TSS=\sum_{i=1}^{N}{\left(y_{\exp,i}-y_{\exp,mean}\right)}^{2}$$ (6)

Equation 6: Definition of the coefficient of determination (R^2^).

**FIGURE 3:**
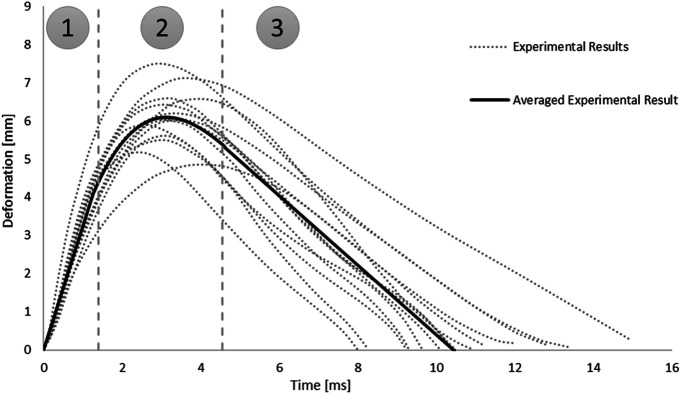
Experimental trajectories including the average response for all impacts of Pellet I on the spinal Cord-pia mater complex divided into three regions: 1) loading, 2) rebound, and 3) unloading (Persson 2009).

In addition, the root-square-mean error (RSME) was calculated for the fitted spinal cord tissue and pia mater material models. Experimental trajectories of the pellets were digitized (Engauge Digitizer v10.6) from the published experimental data (Persson, 2009). To obtain the average trajectory for each pellet, a previously published methodology was adopted (Mattucci and Cronin, 2015). Experimental trajectories of pellets were divided into three regions based on the SCP complex state: 1–loading, 2–rebound, 3–unloading (Figure 3). Loading and unloading regions were fitted with a linear polynomial, whereas a third order polynomial was used to approximate the rebound phase. The average response was determined by calculating pointwise average. Additionally, results of the impact model were compared to the average experimental curve using CORrelation and Analysis method (CORA) (Gehre et al., 2009) (Figure 3), with equally weighted size and shape ratings of 0.5. The size rating compares the area under the curves, while the shape rating compares the trend in slopes between the curves.

## Results

### Spinal Cord and Pia Mater Material Models

**FIGURE 4:**
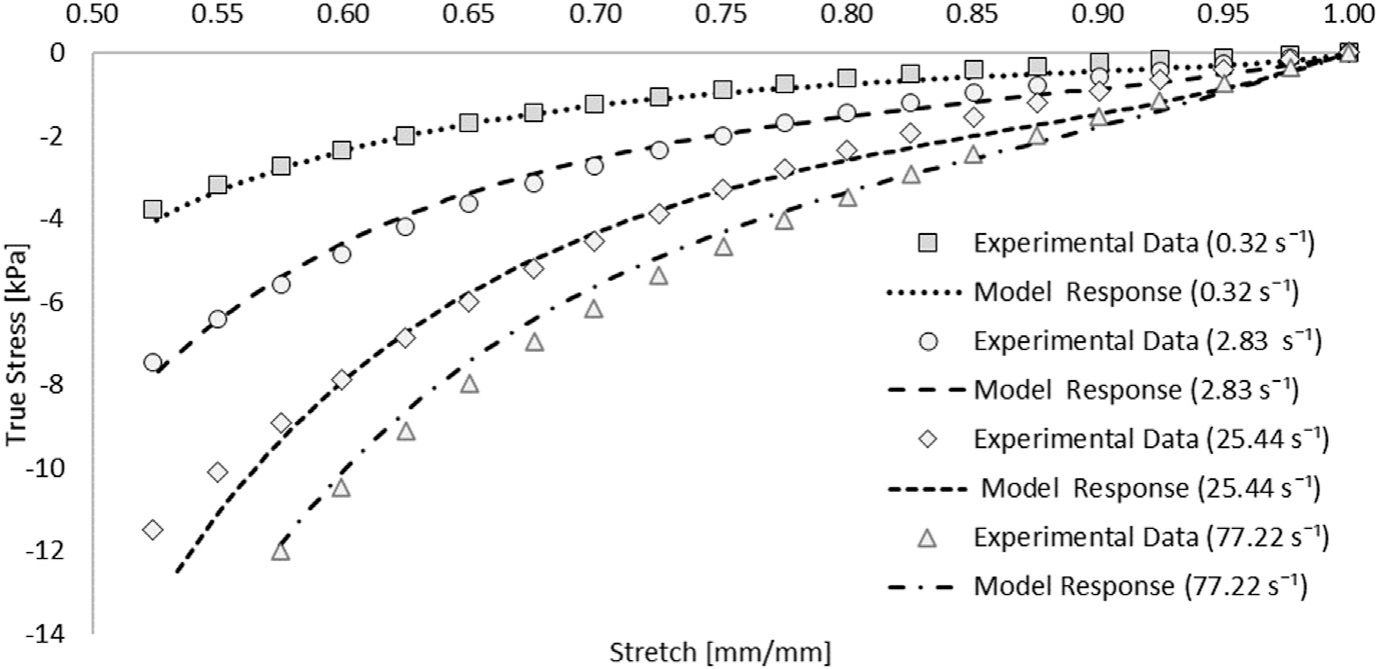
Optimized response of the hyper-viscoelastic Ogden material model in single element test cases compared to unconfined compression test data of the spinal cord tissue (Jannesar et al., 2018).

A one-term Ogden model with quasilinear viscoelasticity (Table 1) provided an excellent fit to all four stress-strain experimental curves: 0.32, 2.83, 25.44, and 77.22 s^−1.^ Single element simulations verified the curve fit (Figure 4). The coefficient of determination (R^2^) ranged from 0.976 to 0.994, with an average RSME of 1.01 kPa [Figure 4].

**TABLE 1:** Summary of the material properties of the spinal cord and pia mater.

Material	Constitutive model	Model parameters and coefficients					References
Spinal cord	Hyperelastic (ogden model) with quasi-linear viscoelasticity	μ = 209 Pa	α = 7.52	ν = 0.499	G_1_ = 0.033	*ß*_1_ = 2 s^−1^	Jannesar et al. (2018)
					G_2_ = 0.296	*ß*_2_ = 13 s^−1^	
					G_3_ = 0.406	*ß*_3_ = 406 s^−1^	
Pia mater	Hyperelastic (ogden model)	μ = 42 kPa	α = 12.58	ν = 0.49			Jin et al. (2006)

**FIGURE 5:**
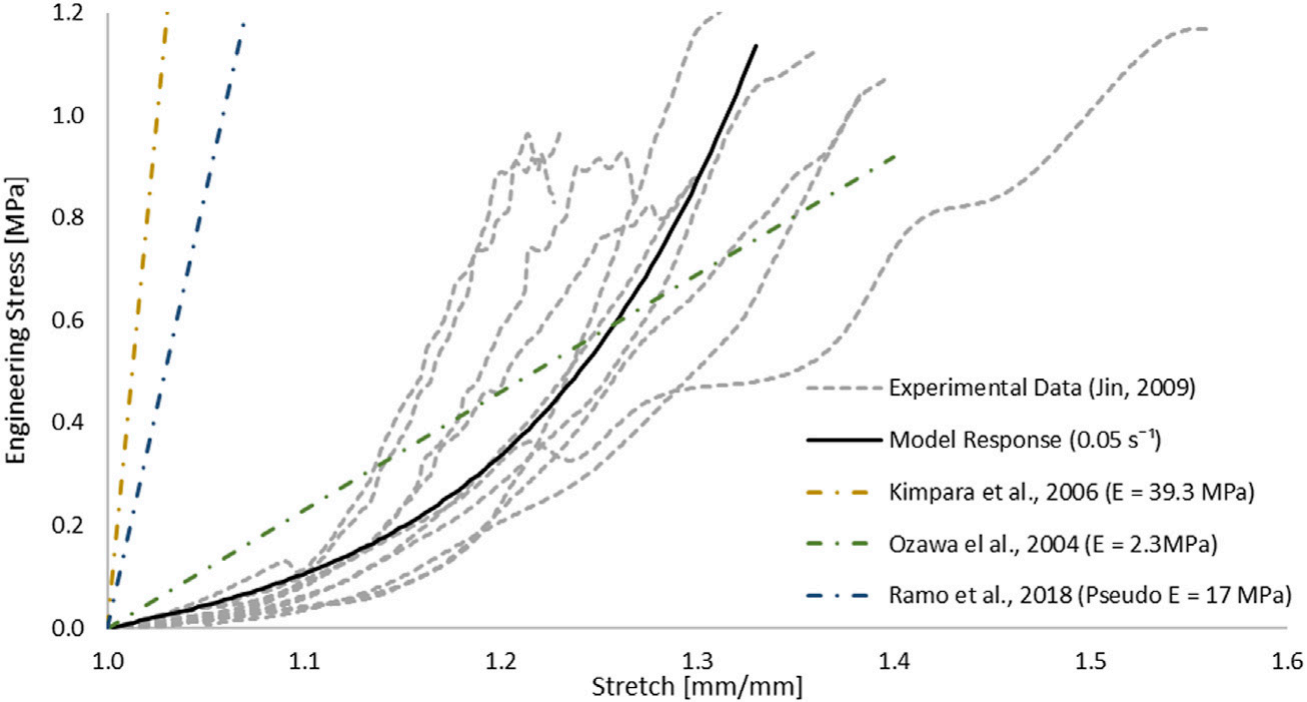
Optimized response of the hyperelastic Ogden material model in a single element test case compared to tensile test data of the pia mater (Jin et al., 2006). Other strain-stress curves with different Young’s modulus reported in studies were presented.

The one-term hyperelastic Ogden representation of the tensile mechanical response of the pia mater was fitted to the average experimental curve (Table 1). The model response fell within the scatter of the experimental data (Figure 5). The RSME for this fit was 29.38 kPa.

### Indentation Test Simulation

**FIGURE 6:**
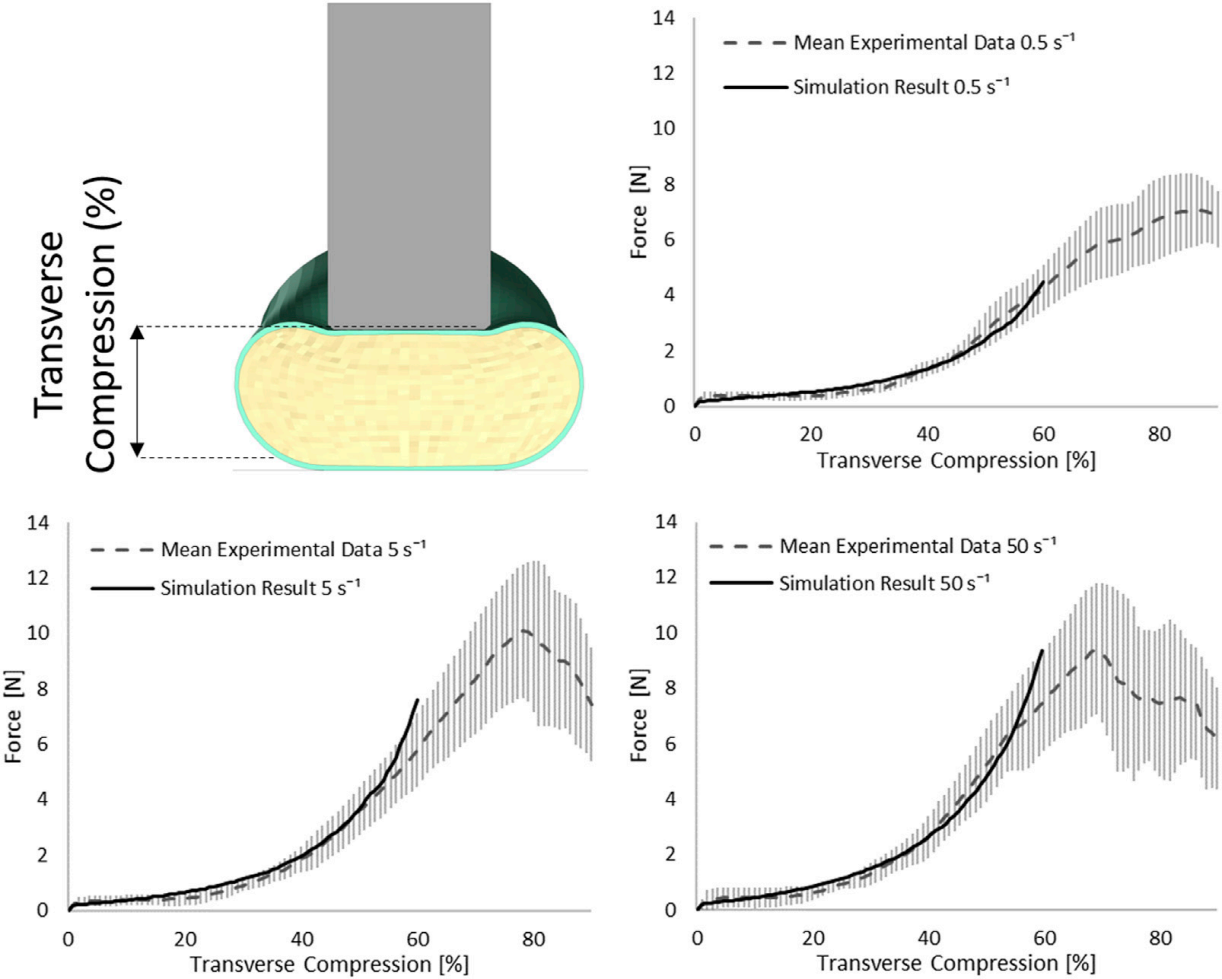
Stress-transverse compression plots for the indentation simulations for three experimental strain rates 0.5, 5, and 50 s^−1^; error bars represent standard deviation of the experimental data (Fradet et al., 2016).

The SCP complex was evaluated in the transverse indentation test for three experimental strain rates: 0.5, 5, and 50 s^−1^. The force acting on the cylinder versus transverse compression of the specimen was compared to reported experimental curves. Simulation results were within one standard deviation for all three strain rates up to 60% of transverse compression of the SCP complex (Figure 6).

### Impact Test Simulation

**FIGURE 7:**
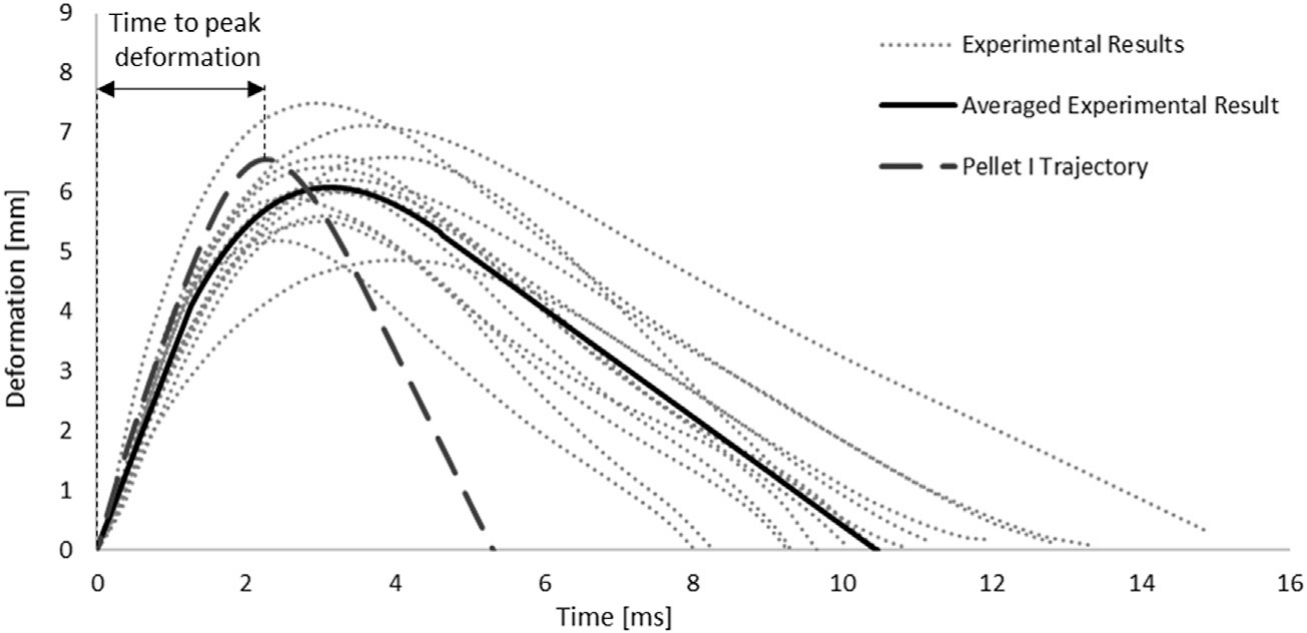
Kinematic response of Pellet I compared to the experimental data (Persson 2009).

The SCP complex FE model was evaluated using experimentally reported data for three transverse pellet impacts on bovine SCP specimens (Persson, 2009; Persson et al., 2009). The model accurately predicted kinematics of the pellets in the loading and rebound phases up to the maximum deformation of the SCP complex (Figure 7). The percentage difference between reported maximum deformation (Persson et al., 2009) and the computed values was 6.63% for Pellet I, 4.00% for Pellet II and 10.17% for Pellet III.

**FIGURE 8:**
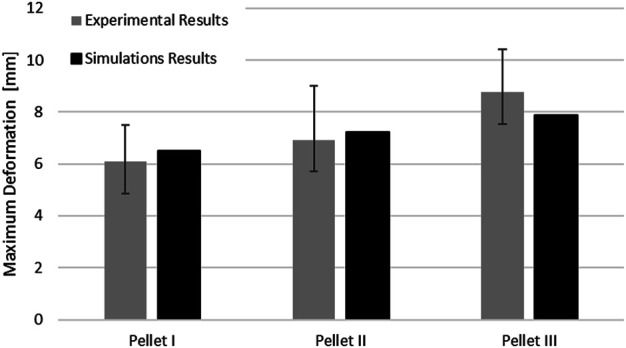
Comparison of the maximum deformation of the SCP complex for three impacts with experimental data.

The maximum deformation of the SCP complex for all three pellets fell within the reported range of the experimental data. The maximum deformation for Pellet I and Pellet II was slightly above the experimental average, whereas the maximum deformation of the Pellet III was just above the lower bound of the reported experimental data (Figure 8).

**FIGURE 9:**
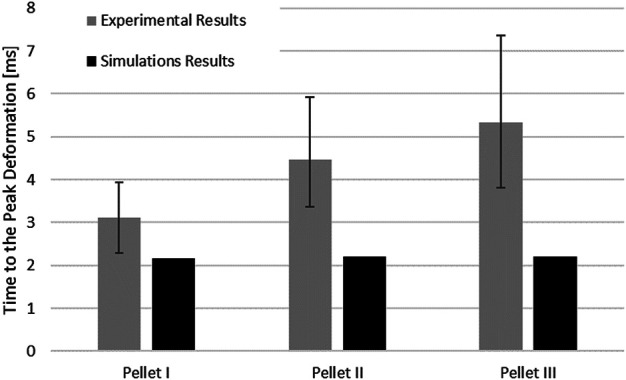
Comparison of the time to the peak deformation for three impacts with experimental data.

**FIGURE 10:**
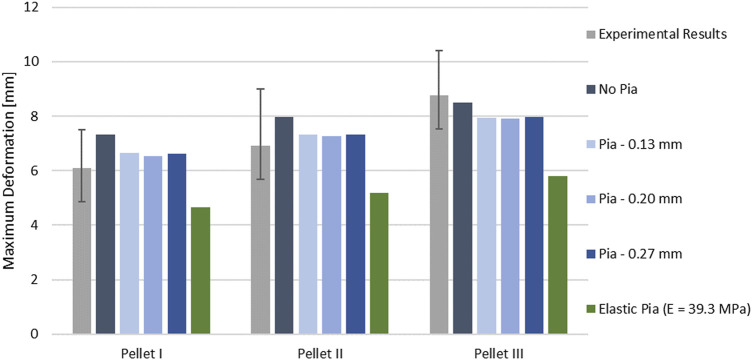
Variation of the peak deformation in the impact model for three pellets with respect to varying thickness of the pia mater.

The kinematics of the pellet during the unload phase happened faster than the experimental data suggested (Figure 7; Figure 9). Results of the impact model were compared to the average experimental curve using CORA. The CORA rating ranged from 0.747 to 0.858, with an average of 0.815. Varying the thickness of the pia mater revealed that the maximum compression of the SCP complex was within 1% across the range of four different values tested (Figure 10). Simplifying the pia mater to linear elastic material properties resulted in maximum deformation below the experimental range. On average the maximum deformation of the SCP complex with elastic pia mater was lower by 38.5% compared to the experimental mean value (Figure 10).

## Discussion

### Material Properties of the SCP Complex

The SCP complex constitutive models were based on measured primate tissue material properties for the spinal cord parenchyma and bovine tissue for the pia mater. The neural system of the non-human primate and human were found to be very similar (Jannesar et al., 2018). Thus, the obtained constitutive models can be taken as a reasonable representation of the expected human neural tissue response under dynamic loading. In experimental studies, other mammalian tissues may be used due to availability and convenience in testing. Several studies have reported similarities between bovine and human spinal cord mechanical properties (Bilston and Thibault, 1995; Oakland et al., 2006). In other studies, porcine tissues were used and have been suggested to be a reasonable surrogate for human tissues (Sparrey and Keaveny, 2011; Shetye et al., 2014; Kim et al., 2018).

The constitutive model parameters identified in this study were not calibrated or altered to improve the expected results of the validation cases. Adjusting or calibrating constitutive model parameters to one specific loading scenario can lead to unphysical response in other types or modes of loading (Cronin, 2011; Yang, 2017). Further, calibration to specific cases can result in material properties outside of the physical bounds for a given material and can lead to changes in FE model behaviour (*e.g.,* contact stiffness, mechanical wave speed propagation). The validation process performed in this study used independent sets of tissue data for fitting material parameters and two independent transverse deformation cases to assess the resulting properties. Applying proposed material parameters (Jannesar et al., 2018) for a first order Ogden quasi-linear viscoelastic model for the spinal cord resulted in significantly lower deformations in the impact model on average by 27% compared to average experimental results, attributed to material constants that differed by as much as one order of magnitude, compared to the values in the present study.

### Transverse Indentation of the SCP Complex

A comparison of the force acting on the cylinder during the indentation experiment showed an excellent match with the average force-compression curves for all experimental strain rates, up to 60% transverse compression of the SCP complex. Above the 60% transverse compression, the simulation results diverged from the experimental results, showing a higher overall stiffness. Local or element-level strains that occur in the spinal cord at such high transverse compression values exceed the range of strains for which the constitutive model was fitted. Fradet et al. (2016) reported failure of the specimen, tearing of the pia mater and expulsion of the white matter. Damage and failure of the material were not incorporated into the fitted constitutive models but should be considered for future models. The mesh sensitivity study revealed that, due to the small size of the indenter (5 mm diameter), accurate representation of the spinal cord deformation required an average spinal cord size mesh of 0.45 mm. However, other studies simulating bone fragments representative for burst fracture have suggested impactor sizes with a diameter of 20 mm (Hall et al., 2006; Jones et al., 2008), which could be modeled with the large mesh sizes proposed in this study.

### Transverse Pellet Impact of the SCP Complex

**FIGURE 11:**
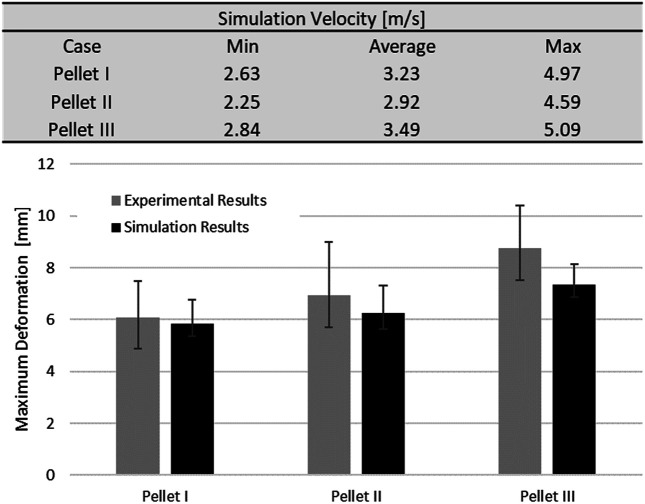
Spinal cord impact model velocity sensitivity study. Values of minimum, maximum and average velocities were calculated from based on initial slope of the experimental data (pellet trajectories).

Results from the impact test simulations were within the experimental variability up to the maximum deformation. On average, the percent difference in maximum deformation between the model and experiments was 7% for all three pellets, but the unloading phase of the FE model was shorter than the experimental unloading phase on average by 60% (5.2 ms) (Figure 7, Figure 9). Moreover, the velocity sensitivity study addresses, in part, the observed variability in the measured spinal cord deformation reported in the experimental data (Figure 11).

**FIGURE 12:**
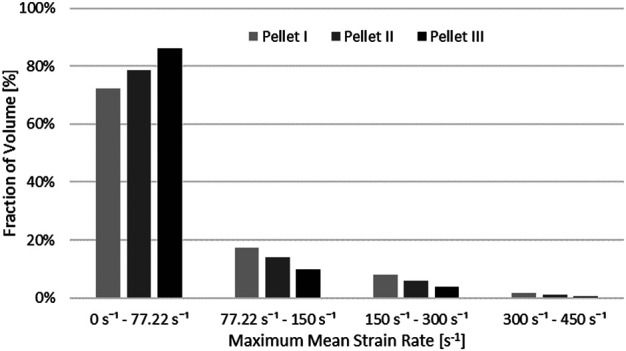
Volume fraction of the spinal cord that was subjected to maximum mean strain rate.

Nervous tissues are composed of 75–77% fluid (Keep et al., 2012; LoPachin et al., 1991). Arguably, during dynamic transverse impact on the SCP complex, highly deformable materials can flow and move out of the impact zone. Inherently a Lagrangian mesh formulation introduces challenges in predicting flow of material. This represents one potential possibility as to why there was a large difference in the unloading phase of the FE model compared to the experimental data. Furthermore, the reported range of strain rates of the tissue-level experimental data of the spinal white matter did not correspond fully to the range of strain rates that was observed during pellet impacts. The maximum mean strain rate that was observed in the spinal cord was measured as a fraction of the volume of the spinal cord (Figure 12). On average, 20.9% (Pellet I–27.4%; Pellet–II 21.4%, Pellet III–14.0%) of the volume of the spinal cord experienced a strain rate above the maximum strain rate (77.22 s^−1^) reported in the tissue-level experiment (Jannesar et al., 2018).

Although the quasi-linear viscoelastic model formulation extrapolates stresses beyond those for which the model was calibrated, it is not clear whether extrapolated stresses are represented faithfully. In summary, it is unknown if the model underestimates or overestimates the extrapolated stresses for strain rates exceeding 77.22 s^−1^. Lastly, Fradet et al. (2016) reported that for the highest transverse compression rate (50 s^−1^) of the SCP complex, damage occurred at compression of 66.9% and for some cases tearing of the pia mater and expulsion of the white matter occurred. Further, some studies have demonstrated that damage of soft tissues is progressive and depends on the applied strain rate (Mattucci et al., 2013). In the impact test simulations, strain rates higher than 50 s^−1^ and similar values of transverse compression (63–76%) were observed. These experimental results suggest that damage could occur in the spinal cord under aggressive impact conditions and future models should consider incorporating damage when sufficient experimental data is available. It can only be hypothesized that damage in the spinal cord tissues could have occurred in the physical test, while no visible tear or failure of the tissue was reported in the experiments (Persson, 2009). Lack of a material damage model could explain the difference between the experiment and FE model in the unloading phase for the pellet impacts (Figure 7).

### Importance of the Pia Mater on Response of the SCP Complex

Previously published studies indicated greater stiffness of the pia mater compared to the spinal cord (Tunturi 1978; Ozawa et al., 2004; Ramo et al., 2018). Further, the reinforcing properties of the pia mater and the influence on the SCP complex were previously acknowledged in the literature (Clarke et al., 2009; Galle et al., 2010). Ozawa et al. (2004) observed that pia mater restores a deformed spinal cord to the original shape after transverse compression and estimated that pia mater was around 460 times stiffer than the spinal cord (Ozawa et al., 2004), in agreement with the material data used in this study (Figure 4; Figure 5). Ramo et al. (2018) recognized that pia-arachnoid mater influenced the longitudinal mechanical response of the SCP complex, despite the relatively low thickness (0.2 mm) of this membrane-like tissue (Watson et al., 2009). Moreover, Ramo et al. (2018) reported that the pia-arachnoid complex comprises up to 5.5% of the transverse area of the SCP complex. In the proposed models in this study, the pia-arachnoid complex area was 7% in the indentation model and varied from 4.54% (0.13 mm) to 9.53% (0.27 mm) for the impact model.

Comparison between the impact models with incorporated pia mater and without showed that the presence of the pia mater reduced the maximum compression of the SPC by 9% on average (Figure 10). The sensitivity study confirmed that pia mater confining effects play a strong role in the spinal cord deformation rather than the pia mater thickness. Jin et al. (2006) reported damage of some pia mater specimens as low as 20% tensile strain (Figure 5), while Kimpara et al. (2004) reported failure of the pia mater under tensile strain in the strain range of 28–48%.

**FIGURE 13:**
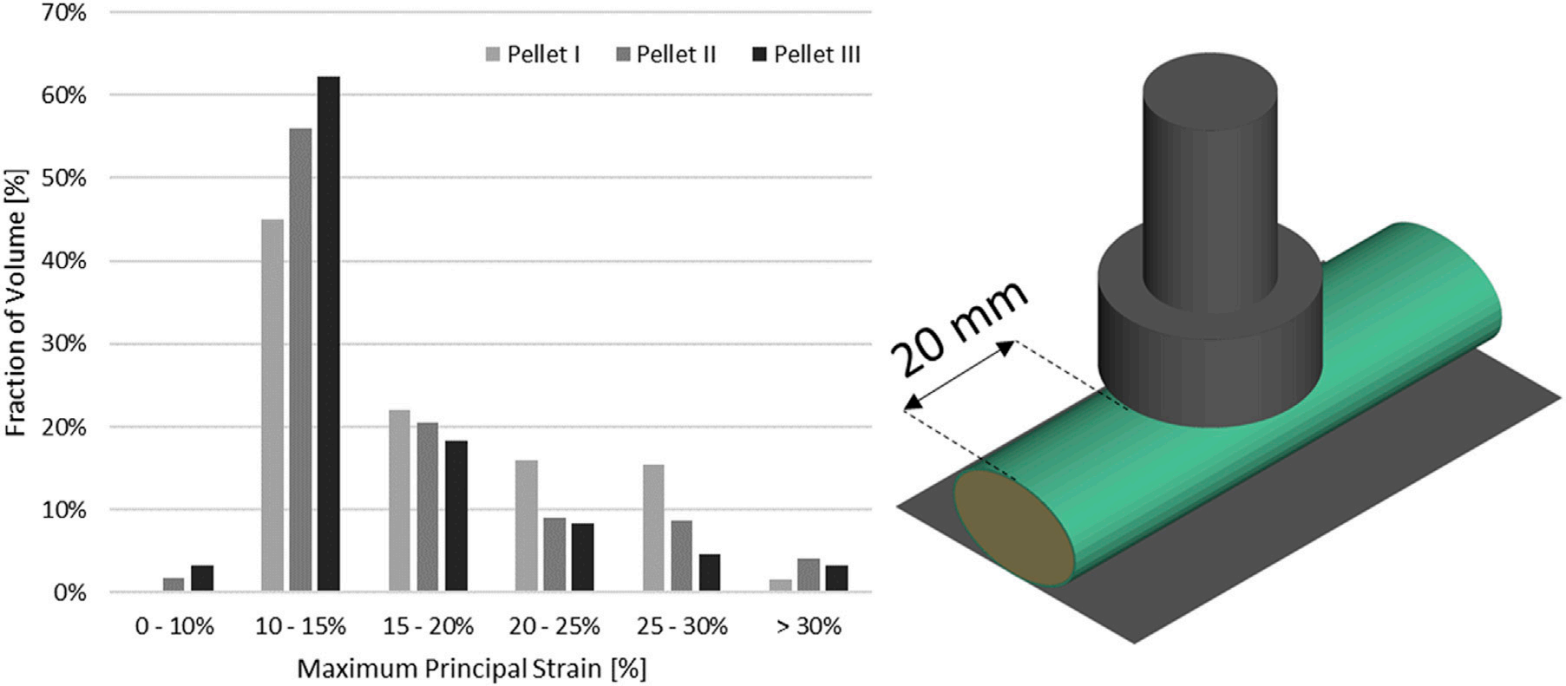
Volume fraction of the pia mater that was subjected to maximum principal strain in the impact model in the vicinity of the impact.

In the vicinity of the impactor (Figure 13), the strains in the pia mater are in the reported failure range, which supports that tearing of the pia mater may have occurred in the impact experiments, resulting in the identified changes to the unloading phase. Further, this agrees with observations that pia mater significantly affects mechanical response of the SCP complex (Jannesar et al., 2016).

Several studies have been conducted to investigate a correlation between spinal cord transverse compression and neurological deficit. Anderson 1985) found that a compression of 50% of the spinal cord leads to neurological sequela. Moreover, Anderson 1985) reported that the deformation rate plays a role, in addition to compression magnitude in SCI severity. Sparrey et al. (2008) performed histological studies on rat spinal cord at two impact velocities (fast and slow), reporting that the velocity of the deformation has a significant effect on spinal cord injury and the related internal hemorrhage. Kearney et al. (1988) observed an absence of neuron transmission after 65% transverse compression of the spinal cord. FE simulation of the impacts of the pellets resulted in transverse compression of the spinal cord by 63, 70, and 76%, for Pellet I, Pellet II, and Pellet III, respectively. Penning et al. (1986) reported neurological sequela when the transverse cross-sectional area of the spinal cord was reduced by 30% or more. In the simulations from the current study, transverse cross-sectional areas of the spinal cord were reduced by 37, 44, and 46% for Pellet I, Pellet II, and Pellet III, respectively. Both injury thresholds (*i.e.,* transverse compression of the spinal cord and reduction of the transverse area) indicate the potential for SCI if the deformations resulting from the pellets impact occurred within a living subject.

### Study Limitations and Conclusion

The presented FE model has several recognized limitations:

1) The obtained hyper-viscoelastic material parameters for the spinal cord parenchyma provided excellent fit to the experimental data (*R* ^2^ = 0.98) and demonstrated good correspondence to independent experimental data through the validation cases. However, it is difficult to absolutely determine if the fitting method used identified the best global material model parameters.
2) The model used a single representation of the transverse dimensions of the SCP complex, and a sensitivity study regarding the expected variation in spinal cord diameter and pia mater thickness was not investigated. In the simulated FE models, the thickness of the pia mater was in the reported range of 130–270 μm (Kimpara et al., 2006).
3) This study focused on examining the effects of material properties that drive the dynamic response of the SCP complex; however, variations in material properties were not investigated and should be considered in future studies. This may have contributed to differences between reported *ex-vivo* experimental data and simulations results.
4) The material models used to represent spinal cord were isotropic.
5) The spinal white matter was calibrated to the experimental data in the strain rate regime up to 77.22 s^−1^. The volume fraction analysis revealed that, on average, 20.9% of the spinal cord volume exceeded a strain rate of 77.22 s^−1^. Additional experimental testing at higher strain rates is needed to assess this limitation.
6) The mechanical properties of the pia mater were obtained from quasi-static experimental data (Jin et al., 2006). Additional test data at elevated deformation rates are needed and should be included in future work.
7) The properties of the spinal cord grey and white matter were lumped together in the present study. The primary intention of the spinal cord FE model developed in this study was to provide a continuum model amenable to implementation in a full HBM for prediction of spinal cord deformation following impact. In this context, modeling the white and gray matter as separate entities would present challenges due to the requisite small element size (0.4 mm) for differentiation of this tissue boundaries in comparison to the relatively large whole-body average element size of 1.8 mm of the HBM (Barker and Cronin, 2021). Moreover, the white matter of the spinal cord has been more frequently characterized in the published data at the strain-rate regime corresponding to the dynamic compression that can be observed during SCI (Sparrey and Keaveny, 2011; Jannesar et al., 2018). Furthermore, previously published studies revealed that separating the spinal cord to white and gray matter had little effect on the resulting deformation under transverse impact loading (Persson et al., 2011). Although modeling the individual white and grey matter tissues may be beneficial in understanding spinal cord injury at a local level, additional work is required to identify specific injury thresholds for the individual spinal cord white and grey matter tissues.

The current study identified and validated material parameters in a continuum-level hyper-viscoelastic constitutive model for the spinal cord tissue and parameters of a hyperelastic constitutive model for the pia mater. Data used in fitting the material parameters were independent of the impact test validation data; once obtained, material model parameters were not changed to improve the outcome of the validation simulations. The pia mater reduced the deformation of the SCP complex resulting from transverse loading. The fitted constitutive models were validated against two tissue-level experiments with an overall good correlation to independent experimental data and could be implemented in a contemporary HBM in the future.

## Data Availability

The original contributions presented in the study are included in the article/Supplementary Material, further inquiries can be directed to the corresponding author.
